# The IAS-MEEG Package: A Flexible Inverse Source Reconstruction Platform for Reconstruction and Visualization of Brain Activity from M/EEG Data

**DOI:** 10.1007/s10548-022-00926-9

**Published:** 2022-12-02

**Authors:** Daniela Calvetti, Annalisa Pascarella, Francesca Pitolli, Erkki Somersalo, Barbara Vantaggi

**Affiliations:** 1 Department of Mathematics, Applied Mathematics and Statistics, Case Western Reserve University, 10900 Euclid Avenue, Cleveland, OH 44106 USA; 2 Istituto per le Applicazioni del Calcolo “M. Picone”, National Research Council, Via dei Taurini 19, 00185 Rome, Italy; 3 Department of Basic and Applied Sciences for Engineering, University of Rome “La Sapienza”, Via Scarpa 16, 00161 Rome, Italy; 4 Dipartimento Metodi e Modelli per l’Economia, il Territorio e la Finanza MEMOTEF, University of Rome “La Sapienza”, Via Castro Laurenziano 9, 00161 Rome, Italy

**Keywords:** Brain activity reconstruction, Bayesian framework, Conditionally Gaussian prior, Sensitivity weighting, Iterative Krylov solver, Sliced visualization

## Abstract

We present a standalone Matlab software platform complete with visualization for the reconstruction of the neural activity in the brain from MEG or EEG data. The underlying inversion combines hierarchical Bayesian models and Krylov subspace iterative least squares solvers. The Bayesian framework of the underlying inversion algorithm allows to account for anatomical information and possible a priori belief about the focality of the reconstruction. The computational efficiency makes the software suitable for the reconstruction of lengthy time series on standard computing equipment. The algorithm requires minimal user provided input parameters, although the user can express the desired focality and accuracy of the solution. The code has been designed so as to favor the parallelization performed automatically by Matlab, according to the resources of the host computer. We demonstrate the flexibility of the platform by reconstructing activity patterns with supports of different sizes from MEG and EEG data. Moreover, we show that the software reconstructs well activity patches located either in the subcortical brain structures or on the cortex. The inverse solver and visualization modules can be used either individually or in combination. We also provide a version of the inverse solver that can be used within Brainstorm toolbox. All the software is available online by Github, including the Brainstorm plugin, with accompanying documentation and test data.

## Introduction

The recording of the extracranial magnetic field, or alternatively, the voltage distribution on the scalp, induced by the concerted firing of bundles of neuron in the brain constitute the data of MagnetoEnchephaloGraphy (MEG) or ElectroEncephaloGraphy (EEG), two modalities for monitoring brain activity in a totally non-invasive manner with an exquisite time resolution in the millisecond range. It is because of its high temporal resolution that the M/EEG modalities are the tools of choice to investigate and localize brain phenomena that occur within a short time interval, such as the initiation of an epileptic seizure.

The challenges in the use of the M/EEG modalities come from the weakness of the signal, and ill-posedness of the inverse problem of mapping the boundary data to the brain region. In particular, the number of M/EEG channels is of the order of one to a few hundreds, while the brain activity is typically represented in the form of an ensemble of current dipoles at a number of locations in the brain ranging in tens of thousands, distributed on the cortex as well as in the internal structures. The difficulties associated with the solution of the M/EEG inverse problem include the low signal-to-noise ratio, the high sensitivity of the solution to the perturbations in the data, and the non-uniqueness of the solution (Hämäläinen et al. 1993; Baillet et al. 2001; Brette and Destexhe 2012), thus requiring that the data is augmented with additional information in the form of a regularization term or a prior. An additional byproduct of the non-uniqueness of the solution is that most classical inversion methods tend to favor sources closer to the sensors, thus giving preference to cortical activity over activation in the deeper brain.

Inverse problems can be recast in the form of Bayesian inference problems, and several algorithms based on Bayesian hierarchical models can be found in the literature (Auranen et al. 2005; Calvetti et al. 2009; Henson et al. 2009, 2010; Kiebel et al. 2008; Lucka et al. 2012; Lopez et al. 2014; Mattout et al. 2006; Nummenmaa et al. 2007a, b; Owen et al. 2012; Sato et al. 2004; Stephan et al. 2009; Trujillo-Barreto et al. 2004; Wipf and Nagarajan 2009; Wipf et al. 2010). The inverse solver algorithm discussed in this paper (Calvetti et al. 2009, 2015) is based on a model where the unknown dipoles are conditionally independent Gaussian random variables whose variances, also unknown, are modeled as random variables following a gamma distribution. The hierarchical structure of the prior, particularly suitable for modeling focal activity patterns, effectively doubles the number of unknowns, as now both the dipoles and their variances need to be estimated. The negative logarithm of the resulting posterior density, or Gibbs energy, has been shown to be globally convex (Calvetti et al. 2009), hence to have a unique minimizer. The Iterative Alternating Sequential (IAS) algorithm computes an approximation of the Maximum a Posteriori (MAP) single estimate of the posterior. Each iteration of the algorithm consists of a sequence of two steps, one requiring the solution of a least squares problem to update the estimate of the dipoles and the other consisting of a formula evaluation to update the estimate of their variances.

In addition to the theoretical advantage of guaranteed unique MAP estimate, the computational advantage of the software lies in the implementation of the optimization algorithm. The two alternating steps consist of a closed formula update of the variances, and a linear least squares problem for updating the dipoles. The latter step, that is potentially time consuming, is implemented in IAS by using a Krylov subspace iterative method based on the Conjugate Gradient for Least Squares (CGLS) with a particular right preconditioner coming from a symmetric factorization of the precision matrix of the prior, therefore referred to as priorconditioner, and a left preconditioner coming from a symmetric factorization of the precision matrix of the noise. Unlike in the traditional use of iterative solvers for linear systems where the prior provides a regularization, the iterative algorithm in IAS solves a reduced low rank linear system informed by the prior, and it is equipped with a suitable early stopping rule, acting as a regularization. This novel approach, the priorconditioned Krylov solver scheme, reduces dramatically the computational cost and has the advantage of converging very fast, typically requiring only few (in the order of tens) matrix-vector products with the lead field matrix, is more than an alternative way of solving a linear system, since the solution with early stopping depends nonlinearly on the data (Calvetti et al. 2018). The subject-specific anatomical information based on the segmented MRI images is encoded in the right preconditioner. Anatomical information about the brain to augment the data has been previously used in MEG algorithms, see, e.g., in Lin et al. (2006).

The popularity of standard public domain M/EEG solvers, e.g., Minimum Norm Estimate (MNE) (Hämäläinen and Ilmoniemi 1984; Lin et al. 2006) and LORETA (Pascual-Marqui 1999) is partly due to the fact that they can be used with minimal user intervention. This observation has motivated our effort to reduce the number of user-provided parameters in the present IAS-MEEG to a minimum, with a clear physical interpretation. The key parameter is an estimate of the signal-to-noise ratio (SNR). Another optional input parameter controls the focality of the solution. In Calvetti et al. (2009), the sparsity-promoting propensity of the IAS algorithm, and its relation to some other algorithms favoring sparse solutions (Gorodnitsky and Rao 1997; Nagarajan et al. 2006; Uutela et al. 1999) was highlighted. In particular, it was shown that the shape paraof the gamma hyperprior distribution controls the sparsity, and, furthermore, in Calvetti et al. (2019), it was shown that at an appropriate limit of the shape parameter, the IAS solution converges towards the weighted Minimum Current Estimate (MCE) (Uutela et al. 1999), providing a good intuition for the role of the shape parameter.

Sensitivity weighting, or depth weighting, has been commonly used in connection with, e.g., MNE and MCE methods to overcome the bias of minimization based methods towards sources closer to the measuring devices. Recently, a proper Bayesian interpretation of the sensitivity weighting has been provided in Calvetti et al. (2019). In the present version of the algorithm, the sensitivity weighting is automatic, arising from a very natural exchangeability argument (Calvetti et al. 2019), requiring that for the given SNR, all dipole configurations with equal cardinality of the support should a priori be equally probable.

In Calvetti et al. (2019), the performance of the IAS-MEEG package for the identification of an active brain region ranging from the cortex to the deep structures of the basal ganglia was systematically compared to that of three other standard inversion methods provided in Brainstorm (Tadel et al. 2011): wMNE (Lin et al. 2006), dSPM (Dale et al. 2000), and sLORETA (Pascual-Marqui 1999; Wagner et al. 2004). Extensive statistical evaluations demonstrated that IAS-MEEG is very well suited for the localization of focal brain activity in both cortical and subcortical regions, and its ability to identify an activation pattern even in the presence of disturbances due to internal brain noise was confirmed.

In this paper we present a standalone software platform for the reconstruction of the neural activity in the brain from MEG or EEG data based on the IAS algorithm, and a visualization of the result as sagittal, coronal and axial slices. The reduction in computing time due to the iterative priorconditioned Krylov subspace least squares solver makes the software particularly suitable for the reconstruction of lengthy time series on standard computing equipment. The algorithm requires minimal user provided input parameters, effectively reduced to an estimate of the signal-to-noise ratio. Optionally, a parameter controlling the focality of the solution, the desired accuracy of the solution, and a parameter related to expected maximal strength of the sources may be entered. The input data consist of the MRI and M/EEG measurements, cleaned of obvious artifacts due, e.g., to heartbeat or eye motion, a discretized source space, and the lead field matrix. The computational procedure consists of the following processing stages: (1) Set up the anatomical prior from information in the MRI data; (2) Initialize the vector of the variances of the hyperpriors for the dipoles according the Bayesian sensitivity weighting formula; (3) Initialize the variances of the dipoles; (4) Update alternatively the estimate of the current dipole moments and their variances until convergence to a specified accuracy has been achieved; (5) Visualize the reconstructed activity.

One of the novelties of the current article is the integration of results from several earlier works, making the model scaling and parameter selection automatic, rendering the necessary user interference minimal, limited to the input of an estimate of the signal-to-noise ratio. However, the algorithm leaves the option to control some of the features such as the parameter controlling the required source focality in the solution. Another novel aspect is the integration of EEG and MEG modalities in the same IAS algorithm, which makes the platform particularly flexible.

The paper is organized as follows. After a brief review of the M/EEG inverse problem from a Bayesian perspective in Section “Materials and Methods”, we recall the main step of IAS algorithm in “The IAS Algorithm” section and discuss the physiological meaning of its parameters. Section 4 describes the lightweight visualization tool that we provide together with the algorithm, and in Section 5 the scripts contained in the IAS-MEEG package are described. Finally, in Section 6 we present some computed examples with synthetic and real data.

## Materials and Methods

### Symbols and Notation

For the convenience of the reader, we begin by stating the assumptions and then establish the notation that we will use in the following sections.

The source space is assumed to have *n* dipoles, some of which have a natural preferred orientation determined by the underlying neuroanatomy. For the *j*-th dipole, $1 \le j \le n$, we denote:

$$\begin{aligned} \begin{array}{ll} \textbf{r}_j \in {{\mathbb {R}}}^3 &{} \hbox {point coordinates}, \\ \textbf{q}_j \in {{\mathbb {R}}}^3 &{} \hbox {dipole moment}, \\ \mathbf {\nu }_j \in {{\mathbb {R}}}^3 &{} \hbox {preferred dipole direction}, \\ \theta _j \in {{\mathbb {R}}}&{} \hbox {dipole variance}, \\ \theta ^*_j, \beta _j \in {{\mathbb {R}}}&{} \hbox {hyperparameters}, \\ {{\textsf{C}}}_j \in {{\mathbb {R}}}^{3\times 3} &{} \hbox {local anatomical prior covariance}.\\ \end{array} \end{aligned}$$

When needed, the dipole variables are collected in the vectors:

$$\begin{aligned} \begin{array}{lcl} R &{}=&{} [\textbf{r}_1, \ldots , \textbf{r}_n ]\in {{\mathbb {R}}}^{3n},\\ Q &{}=&{} [\textbf{q}_1, \ldots , \textbf{q}_n ]\in {{\mathbb {R}}}^{3n},\\ N &{}=&{} [\mathbf {\nu }_1, \ldots , \mathbf {\nu }_n ]\in {{\mathbb {R}}}^{3n},\\ \varTheta &{}=&{} [\theta _1, \ldots , \theta _n ]\in {{\mathbb {R}}}^n,\\ \varTheta ^* &{}=&{} [\theta ^*_1, \ldots , \theta ^*_n ]\in {{\mathbb {R}}}^n. \\ \end{array} \end{aligned}$$

The local anatomical prior matrices are used to construct the block diagonal matrix

$$\begin{aligned} {{\textsf{C}}}= \textrm{diag}({{\textsf{C}}}_1, \ldots ,{{\textsf{C}}}_n ) \in {{\mathbb {R}}}^{3n\times 3n}, \end{aligned}$$

that admits a Cholesky factorization as

$$\begin{aligned} {{\textsf{C}}}={{\textsf{D}}}^{{\textsf{T}}}{{\textsf{D}}}, \end{aligned}$$

where ${{\textsf{D}}}$ is an upper triangular matrix. In the following we will also use the matrix

$$\begin{aligned} {{\textsf{C}}}_\theta = \textrm{diag}(\theta _1{{\textsf{C}}}_1, \ldots ,\theta _n{{\textsf{C}}}_n ) \in {{\mathbb {R}}}^{3n\times 3n}, \end{aligned}$$

and its factorization

$$\begin{aligned} {{\textsf{C}}}_\theta ={{\textsf{D}}}_{\theta }^{{\textsf{T}}}{{\textsf{D}}}_{\theta }. \end{aligned}$$

In the solution of the M/EEG inverse problem we use the notation

$$\begin{aligned} \begin{array}{ll} {{\textsf{M}}}\in {{\mathbb {R}}}^{m\times 3n} &{} \hbox {the lead field matrix}, \\ B \in {{\mathbb {R}}}^{m\times t} &{} \hbox {a dataset of t time slices}, \\ \varepsilon \in {{\mathbb {R}}}^{m} &{} \hbox {the noise vector}, \\ \varvec{\Sigma }\in {{\mathbb {R}}}^{m\times m} &{} \hbox {the noise covariance matrix}. \\ \end{array} \end{aligned}$$

We will denote a time slice of data by the vector $b \in {{\mathbb {R}}}^m$, with *m* the number of M/EEG sensors.

Finally, for a vector $V \in {{\mathbb {R}}}^n$ and a matrix ${{\textsf{A}}}\in {{\mathbb {R}}}^{n \times n}$ we use the norms:

$$\begin{aligned} \Vert V\Vert ^2 = V^{{\textsf{T}}}\, V, \end{aligned}$$

and

$$\begin{aligned} \Vert V\Vert ^2_{{\textsf{A}}}= V^{{\textsf{T}}}{{\textsf{A}}}^{-1} V. \\ \\ \end{aligned}$$

### The Bayesian Hierarchical Model

The cortical and sub-cortical surfaces of the brain are extracted from the MR images of the subject under study and discretized in a regular triangulation whose nodes form the source space. A current dipole is located in each point $\textbf{r}_j$, $1\le j \le n$, of the source space. Let $\textbf{q}_j$, $1\le j \le n$, be the moment of the *j*-th dipole. The primary unknown is the current dipole moment vector $Q=[\textbf{q}_1,\ldots ,\textbf{q}_n]$. Each dipole has a preferred direction $\mathbf {\nu }_j$ that can be extracted from the MRI. The local prior variance of the amplitude of each dipole $\theta _j$ is modeled as a random variable following the Gamma distribution with hyperparameters $(\beta _j,\theta _j^*)$:

$$\begin{aligned} \theta _j \sim \pi _{\textrm{hyper}}^j(\theta _j \mid \theta ^*_j, \beta _j)={\varGamma }(\theta _j^*,\beta _j) \propto \theta _j^{\beta _j-1}\textrm{exp}\left( -\frac{\theta _j}{\theta _j^*}\right) . \end{aligned}$$

The observation vector $b\in {{\mathbb {R}}}^m$ and the dipole moment vector $Q \in {{\mathbb {R}}}^{3n}$ are assumed to be linearly related:

1 $$\begin{aligned} b = {{\textsf{M}}}\, Q + \varepsilon , \end{aligned}$$

where ${{\textsf{M}}}\in {{\mathbb {R}}}^{m\times 3n}$ is the lead field matrix and $\varepsilon \in {{\mathbb {R}}}^m$ is the observation noise vector.

Modeling the noise term as a zero mean Gaussian random variable, $\varepsilon \sim {{\mathcal {N}}}(0,\varvec{\Sigma })$, where $\varvec{\Sigma }\in {{\mathbb {R}}}^{m\times m}$ is the noise covariance matrix, the likelihood density of *b* conditional on *Q* can be written as

$$\begin{aligned} \pi (b\mid Q)\propto \textrm{exp} \left( -\frac{1}{2} \Vert b -{{\textsf{M}}}\,Q\Vert ^2_{\varvec{\Sigma }}\right) . \end{aligned}$$

We define the Bayesian hierarchical prior model of the activity in position $r_j$ as

$$\begin{aligned} \pi _{\textrm{prior}}^j(\textbf{q}_j\mid \theta _j)\sim {\mathcal N}(0,\theta _j {{\textsf{C}}}_j)\propto \textrm{exp}\left( - \frac{\Vert \textbf{q}_j\Vert ^2_{{{\textsf{C}}}_j}}{2\theta _j} - \frac{3}{2}\log \theta _j\right) , \end{aligned}$$

where ${{\textsf{C}}}_j\in {{\mathbb {R}}}^{3\times 3}$ is the local anatomical prior matrix

$$\begin{aligned} {{\textsf{C}}}_j = \mathbf {\nu }_j\mathbf {\nu }_j^{{\textsf{T}}}+ \delta (\mathbf {\xi }_j\mathbf {\xi }_j^{{\textsf{T}}}+ \mathbf {\zeta }_j\mathbf {\zeta }_j^{{\textsf{T}}}), \quad 0<\delta <1, \end{aligned}$$

and $(\mathbf {\xi }_j,\mathbf {\zeta }_j,\mathbf {\nu }_j)$ is a local orthonormal frame at $\textbf{r}_j$.

Assuming a priori that the dipoles are conditionally independent leads to a conditionally Gaussian prior model

$$\begin{aligned} \pi _{\textrm{prior}}(Q \mid \varTheta ) = \prod _{j=1}^n\pi _{\textrm{prior}}^j(\textbf{q}_j\mid \theta _j), \end{aligned}$$

with the hyperprior model

$$\begin{aligned} \pi _{\textrm{hyper}}(\varTheta \mid \varTheta ^*,\beta ) = \prod _{j=1}^n \pi _{\textrm{hyper}}^j(\theta _j\mid \theta _j^*,\beta _j). \end{aligned}$$

It follows from Bayes’ theorem that the posterior distribution is of the form

$$\begin{aligned} \begin{array}{lcl} \pi (Q,\varTheta \mid b,\varTheta ^*,\beta ) &{}\propto &{} \pi (b\mid Q) \, \pi (Q,\varTheta \mid \varTheta ^*,\beta ) \\ \\ &{} \propto &{} \displaystyle \textrm{exp}\bigg (-\frac{1}{2} \Vert b -{{\textsf{M}}}\, Q\Vert ^2_{\varvec{\Sigma }} -\frac{1}{2}\sum _{j=1}^n \frac{\Vert \textbf{q}_j\Vert _{{{\textsf{C}}}_j}^2}{\theta _j} \\ \\ &{} &{} \displaystyle +\sum _{j=1}^n\left( \beta _j -\frac{5}{2}\right) \log \theta _j - \sum _{j=1}^n\frac{\theta _j}{\theta _j^*} \bigg ). \end{array} \end{aligned}$$

The Maximum A Posteriori (MAP) estimate of both the dipole moment vector *Q* and corresponding variance vector $\varTheta$ is obtained minimizing the energy function2by the IAS algorithm. In fact, the minimization with respect to *Q* affects only part (*a*) and reduces to a quadratic minimization problem that can be solved very efficiently using a priorconditioned CGLS algorithm, while the minimization with respect to the hyperparameters $\theta _j$ affects only part (*b*) and admits a solution in closed form.

## The IAS Algorithm

**Fig. 1:**
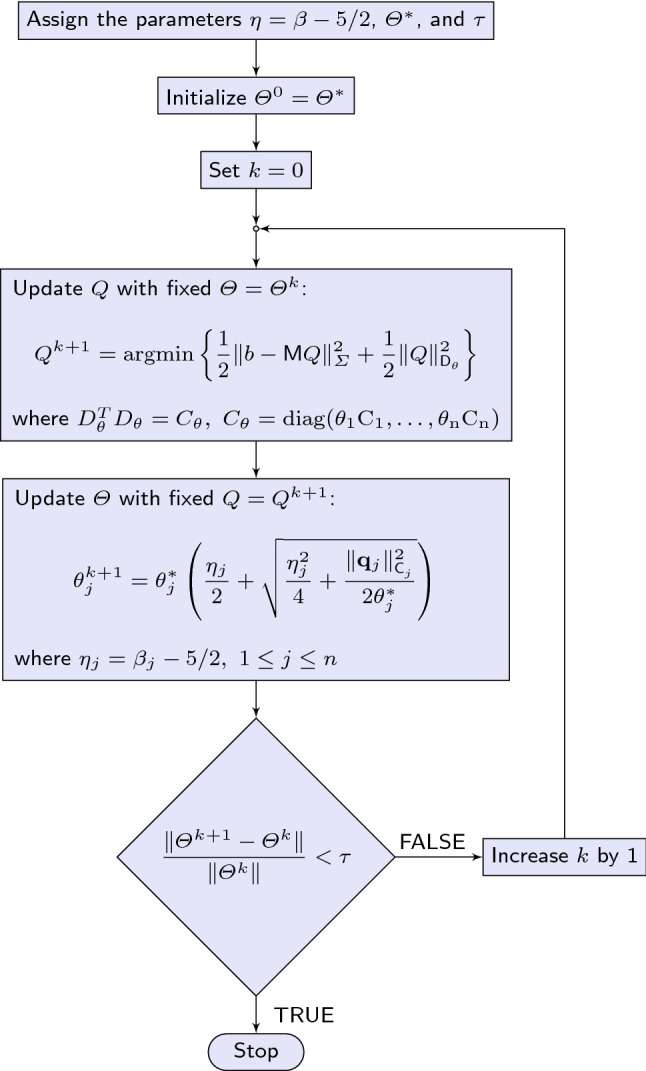
The IAS flowchart (left) and the IAS algorithm (right). The outer loop on *k* is performed through statements 6–25; the inner loop (statements 12–20) solves the minimization problem for $Q^{k+1}$ by the preconditioned CGLS algorithm; the estimated $Q^{k+1}$ is computed by statement 21; the estimated $\varTheta ^{k+1}$ is computed by statements 22–24. Observe that while the IAS algorithm requires the vector $\varTheta ^*$ as input, the user specifies only the signal-to-noise ratio, and $\varTheta ^*$ is automatically generated based on the SNR-exchangeability argument. The parameters $\eta$ and $\tau$ have default values, and specifying them by the user is optional

### The Algorithm

The IAS algorithm minimizes the energy function ${{\mathcal {E}}}(Q,\varTheta )$ by proceeding through a sequence of iteration steps alternating the minimization with respect to *Q* and the minimization with respect to $\varTheta$. The algorithm is initialized by assigning to $\varTheta$ a given value, for instance, $\varTheta ^*$. The first step is to minimize part (*a*) of the energy function (2) with fixed $\varTheta =\varTheta ^*$. The MAP estimate of *Q* given $\varTheta =\varTheta ^*$ is obtained by solving the minimization problem

3 $$\begin{aligned} Q_{\textrm{est}} = \textrm{argmin}\bigg \{ \frac{1}{2} \Vert b - {{\textsf{M}}}Q\Vert _{\varvec{\Sigma }}^{2} + \frac{1}{2} \Vert Q\Vert _{{{\textsf{D}}}_{\theta }}^2\bigg \}. \end{aligned}$$

The minimizer can be found by solving an associated linear system in the least squares sense, and an efficient implementation can be done using a priorconditioned CGLS algorithm, see (Calvetti et al. 2015) for details. Once $Q_{\textrm{est}}$ has been computed, the updated value of $\varTheta$ is obtained minimizing part (*b*) of (2) keeping $Q=Q_{\textrm{est}}$ fixed,

$$\begin{aligned}{} & {} \varTheta _{\textrm{est}} = \textrm{argmin}\left\{ \displaystyle \sum _{j=1}^n\frac{\Vert \textbf{q}_j\Vert ^2_{{{\textsf{C}}}_j}}{\theta _j} \right. \\{} & {} \quad \left. \displaystyle -2 \sum _{j=1}^n\left[ \left( \beta _j - \frac{5}{2}\right) \log \theta _j -\frac{\theta _j}{\theta _j^*}\right] \right\} . \end{aligned}$$

This problem has the analytical solution

4 $$\begin{aligned} \theta _{\textrm{est},j} = \theta _j^*\left( \frac{\eta _j}{2} + \sqrt{ \frac{\eta _j^2}{4} + \frac{\Vert \textbf{q}_j\Vert _{{{\textsf{C}}}_j}^2}{2\theta _j^*}} \right) , \; 1\le j\le n. \end{aligned}$$

where $\eta _j = \beta _j -5/2$. Subsequently, a new estimate of *Q* is obtained by solving problem (3) again with $\varTheta =\varTheta _{\textrm{est}}$. Thus, the minimization problems (3) and (4) are solved alternatingly until a convergence criterion is met. The flowchart of the IAS algorithm is shown in the left of Fig. 1 with the pseudocode of the algorithm shown in the right.

### Choice of the Parameters

While the IAS algorithm depends on two parameter vectors, the shape parameter vector $\beta$ and the scaling parameter vector $\varTheta ^*$, the user needs only to enter an estimate for the signal-to-noise ratio. Internally to the algorithm, the scaling parameter is automatically computed, and the shape parameter is in practice reduced to a single scalar input that can be set at a default value. The description is given below.

The shape parameters $\beta _j > 5/2$, $1 \le j \le n$, control the focality of the reconstructed sources: values close to the lower bound favor sparse solutions while larger values favor distributed solutions. For the sake of simplicity, we assume that the same value of $\beta _j = \beta$ is used for all dipoles in the source space. Recalling that $\eta _j = \beta _j -5/2$, $1 \le j \le n$, we set $\eta _j =\eta$, $1 \le j \le n$. Reasonable values of $\eta$ are in the interval [0.0001, 0.1]: when $\eta =0.0001$ the algorithm favors current density estimates with narrow support, while for $\eta =0.1$ the support of the estimated density is wider.

The scaling parameters $\theta _j^*$, $1 \le j \le n$, are related to the expected value of the variance $\theta _j$ of the *j*-th dipole. By using a Bayesian argument that is based on requiring exchangeability of dipoles to explain the signal-to-noise ratio shows that $\theta _j^*$ needs to be chosen inversely proportional to the sensitivity with respect to the *j*-th dipole. Its value can be evaluated explicitly by the formula

$$\begin{aligned} \theta _j^* = \frac{P - \textrm{trace}(\varvec{\Sigma }) }{\beta \Vert {{\textsf{M}}}_j \, {{\textsf{C}}}_j ^{1/2}\Vert ^2_F}, \quad 1 \le j \le n, \end{aligned}$$

where *P* is the power of the signal, $\varvec{\Sigma }$ is the noise covariance matrix, ${{\textsf{M}}}_j\in {{\mathbb {R}}}^{m \times 3}$ is the local lead field matrix and $\Vert \cdot \Vert _F$ denotes the Frobenius norm (Calvetti et al. 2019). Recalling the definition of the signal-to-noise ratio (SNR) as

$$\begin{aligned} SNR = \frac{\text{ signal } \text{ power }}{\text{ noise } \text{ power }} = \frac{P}{\textrm{trace}(\varvec{\Sigma })}, \end{aligned}$$

we arrive at the formula

$$\begin{aligned} \theta _j^* = \frac{P}{\beta \Vert {{\textsf{M}}}_j \, {{\textsf{C}}}_j ^{1/2}\Vert ^2_F}\left( 1 - \frac{1}{SNR}\right) , \quad 1 \le j \le n. \end{aligned}$$

In Calvetti et al. (2019), the formula was related to the estimate of active focal sources via the exchangeability argument.

### Time Series

In case of time dependent data, the IAS algorithm is applied to each individual time slice. The time slices can be processed individually, or treated as a time series. In the latter case, the hyperparameter vector $\varTheta$ is initialized to $\varTheta ^*$ in the first time step, and set equal to the final estimate in the previous time step in each subsequent time steps. The rationale for the dynamic initialization of $\varTheta$ is that since we do not expect significant changes in the current density from one time instance to the next, it is reasonable to assume the variance $\varTheta$ not to vary much either, thus making the variance at time step *t* a good estimate of the variance at time step $t+1$. Computed examples show that in this way, the outer iteration loop typically requires only a few iterations to reach convergence.

## Visualization of the Activity Map

**Fig. 2:**
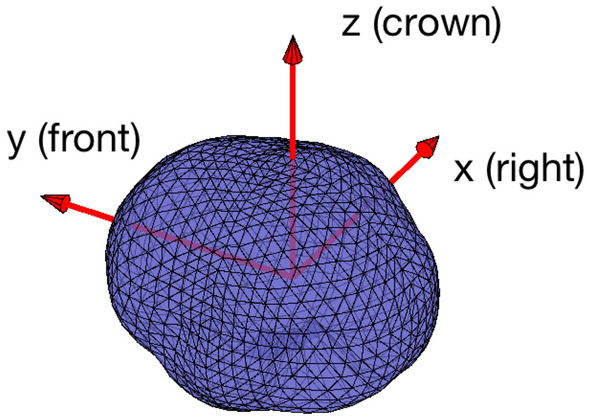
Orientation of the default coordinate axes

To provide a standalone platform, the software is equipped with a computationally lightweight visualization tool based on basic graphic packages of Matlab. The visualization provides a sliced view of the reconstructed activity in three standard orthogonal anatomical planes, axial, coronal, and sagittal views. Given the source space in terms of the projections onto the three principal directions,

$$\begin{aligned} S = [\textbf{r}_1,\textbf{r}_2,\ldots ,\textbf{r}_n], \quad \textbf{r}_j = (x_j,y_j,z_j), \end{aligned}$$

where the default right handed Cartesian coordinates axes are assumed to be in the order $x =\text{ right }$, $y = \text{ front }$ and $z = \text{ crown }$ (see Fig. 2).

The sliced visualization algorithm subdivides the source space in the chosen direction into ten layers. More precisely, for instance in the axial projections, the vertices are parceled as

$$\begin{aligned} S_\ell ^{\textrm{axial}} = [ \textbf{r}_j \mid z_{\ell -1}\le z_j<z_{\ell }], \quad 1\le \ell \le 10, \end{aligned}$$

where $z_\ell = z_{\textrm{min}} +\ell (z_{\textrm{max}} - z_{\textrm{min}})/10$ with $\ell = 0,1,\ldots , 10$, and $z_{\textrm{min}}$ and $z_{\textrm{max}}$ define the extremal values of the source space components $z_j$. Given the IAS-MEEG reconstruction of the brain activity, the visualization algorithm shows the intensities $a_j = \Vert \textbf{q}_j\Vert$ of the dipoles in each parcel, $\textbf{r}_j \in S_\ell ^{\textrm{axial}}$, by plotting the projection $(x_j,y_j)$ as a dot in two dimensions, encoding the intensity in the form of a color map.

In addition to the three sliced views of the full brain, the visualization software allows the user to select a point inside the brain, and produce an activity plot in the form of the single axial, coronal, and sagittal slices containing the selected point. This view is particularly useful if synthetic data corresponding to a focal source are used to monitor how well the algorithm is able to localize a focal source.

**Fig. 3:**
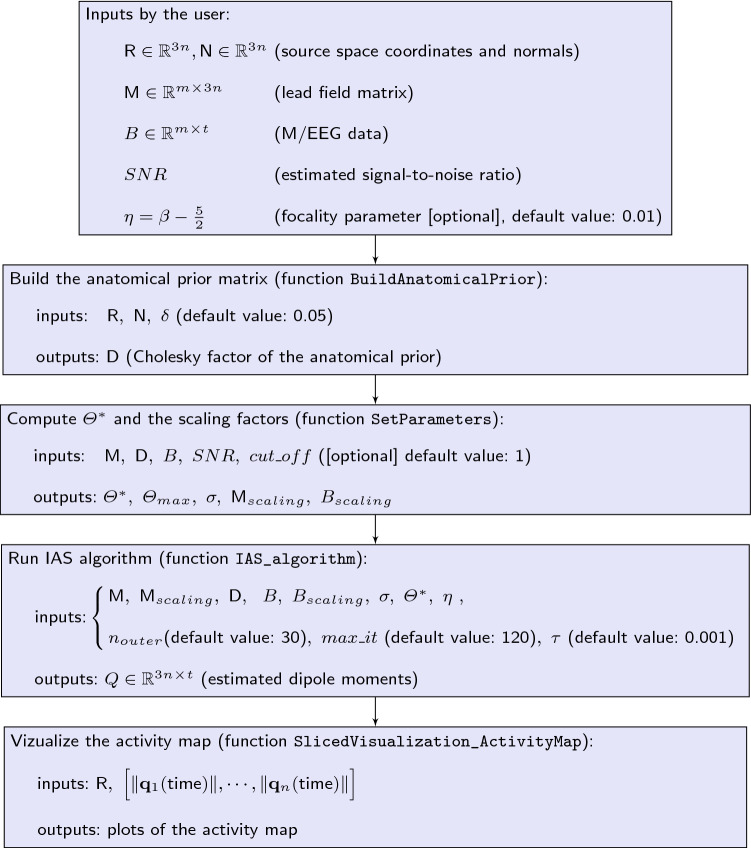
The IAS-MEEG Package. The functions BuildAnatomicalPrior, SetParameters, IAS_algorithm perform the IAS algorithm. The function SlicedVisualization_ActivityMap displays the reconstructed activity map

## IAS-MEEG Package

The IAS-MEEG Package is available at https://github.com/IAS-code/IAS-MEEG and distributed under a Berkeley Software Distribution (BSD) license. The documentation can be found at https://ias-code.github.io/IAS-MEEG/index.html.

The package is coded in Matlab using only basic commands and does not require any of the Matlab toolboxes.

The package comprises four functions:

1. BuildAnatomicalPrior: This function generates the anatomical prior that favors dipoles in the preferred direction. The inputs are: the coordinates of the points in the source space; the normal vectors extracted from the MRI. The user can choose the value of $\delta$, the relative variance of the components of the dipoles. The default value is 0.05. The output is the matrix ${{\textsf{D}}}\in {{\mathbb {R}}}^{3n\times 3n}$, that is the Cholesky factor of the anatomical prior covariance matrix.
2. SetParameters: This function scales the lead field matrix and the data, adjusts the truncation of the sensitivities, and returns the scaling vector $\varTheta ^*$ together with an estimate for the standard deviation of the noise for whitening. The inputs are: the lead field matrix; the Cholesky factor of the anatomical prior covariance as computed by BuildAnatomicalPrior; a small set of the data; the estimated signal-to-noise ratio; the percentage of the highest $\theta ^*_j$ values to be removed (optional: by default no truncation). Removed values can be checked by graphical inspection asking the function to produce plots. The outputs are: the $\varTheta ^*$ vector; the cut-off value used to remove the higher values of $\varTheta ^*$; the standard deviation of the scaled noise; the scaling factor for the lead field matrix; the scaling factor for the data.
3. ${\texttt {IAS}}\_{\texttt {algorithm}}$: This function solves the M/EEG inverse problem by the IAS algorithm described in “The Algorithm section. The inputs are: the lead field matrix; the dataset; the Cholesky factor of the anatomical prior covariance matrix, evaluated in BuildAnatomicalPrior; the parameters, evaluated in SetParameters; the value of $\eta$ for selecting the focality of the reconstructed activity (default value: 0.01; choose 0.001 for focal sources). The user can choose the maximum number of iterations in the outer and inner loops, $n_{outer}$ and $max_{it}$, respectively, and the tolerance $\tau$ used in the stopping criterion for $\varTheta$. Default values are: $n_{outer}$=30, $max\_{it}$=120 and $\tau$ = 0.01. The output is the estimated dipole moment vector for each point of the source space, and a diagnostics matrix indicating the number of inner iterations for each outer iteration, and the relative change in $\varTheta$ when tolerance is reached.
4. ${\texttt {SlicedVisualization}}\_{\texttt {ActivityMap}}$: This function plots the activity map, that is the estimated current intensity of each dipoles (cf. “Visualization of the Activity Map” section). The inputs are: the coordinates of the points in the source space; the estimated intensity vector. The outputs are the plots of the dipole intensity for different sections (axial, coronal and sagittal sections).

In addition, a visualization algorithm is included, which is particularly useful when simulated data are used.

4’: ${\texttt {SingleSliceVisualization}}\_{\texttt {ActivityMap}}$: This function plots three sections (axial, coronal and sagittal sections) of the activity map passing through a given point in space. The inputs are: the coordinates of the points in the source space; the estimated intensity vector; the point at which the three views intersect. Optional inputs include the coordinate system specification and the type of marker indicating the intersection of the three views in the plot.

To solve the M/EEG inverse problem by the IAS-MEEG algorithm the four functions must be called sequentially as shown in the flowchart in Fig. 3. The first box of the flowchart shows all the input necessary to run the IAS algorithm. The lead field matrix ${{\textsf{M}}}$, the source space ${{\textsf{R}}}$ and the corresponding normal orientation ${{\textsf{N}}}$ that need to be given in input can be computed using an available software package (e.g. Brainstorm or Fieldtrip). These inputs are passed as Matlab variables to the different functions as specified in the correspondig box of the flowchart in Fig. 3. We also assume that the input data matrix *B* contains M/EEG data that were already preprocessed (e.g. filtering, artifact removal) and is also passed as Matlab variable.

### Brainstorm Plugin

The IAS algorithm is also available as a Brainstorm plugin, allowing its integration into the Brainstorm workflow. The added value of this integration is that it makes it possible for a user to perform all analysis steps, such as data preprocessing and visualization, within the same toolbox. The three main functions of IAS code, BuildAnatomicalPrior, SetParameters and ${\texttt {IAS}}\_{\texttt {algorithm}}$ are integrated into the single function ${\texttt {process}}\_{\texttt {IAS}}$ available in the IAS-MEEG package. To be used within Brainstorm this function must be copied in the Brainstorm folder

**Fig. 4:**
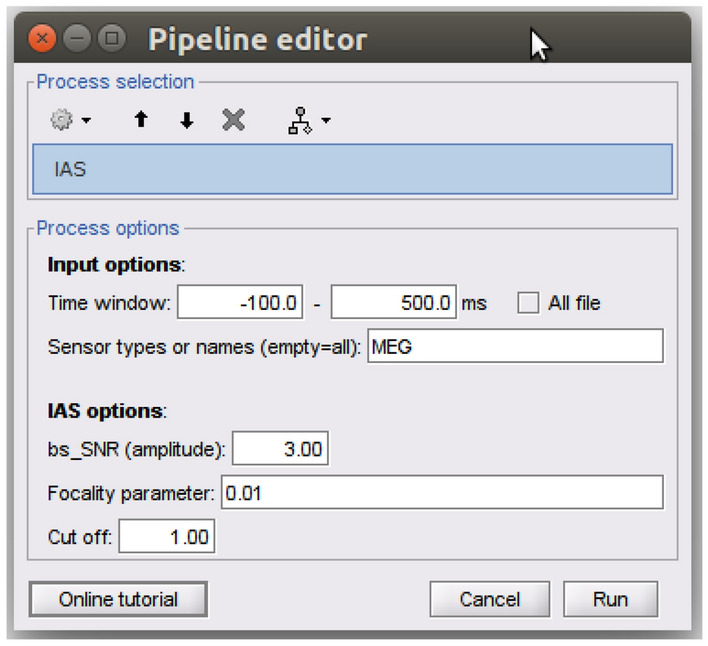
Main window of IAS process within Brainstorm toolbox

brainstorm3/toolbox/process/functions and launched as a process. Figure 4 shows the main window for running IAS within Brainstorm with the input parameters to be provided by the user.

## Results

### MEG Simulated Data

**Fig. 5:**
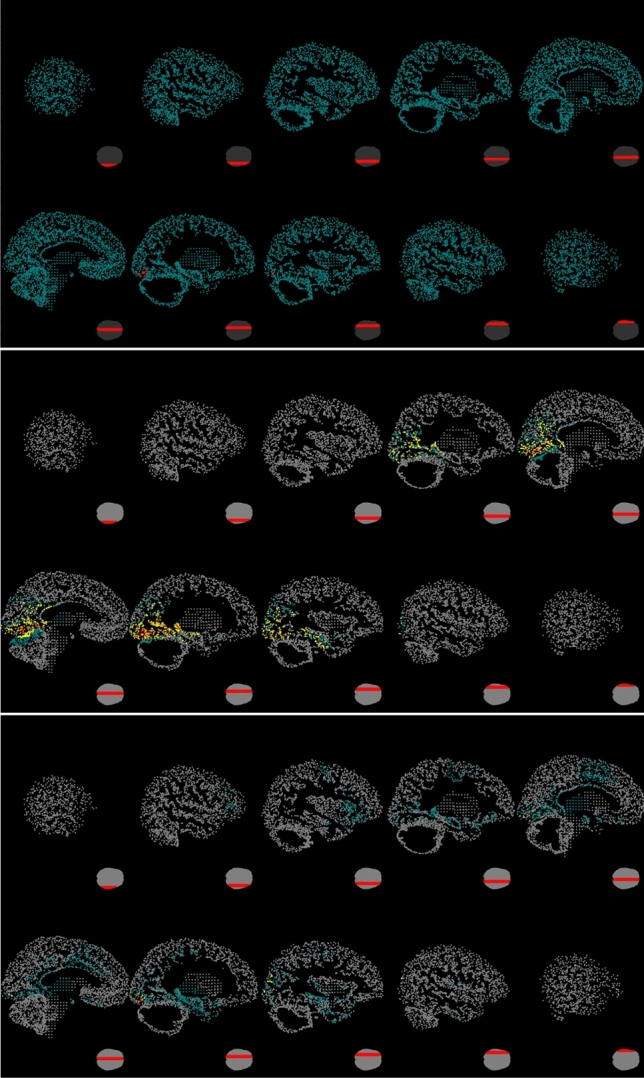
Top: The patch (red dots) used to generate the simulated data. Middle: The estimated neural activity for simulated data when $\eta =0.1$. Bottom: The estimated neural activity for simulated data when $\eta =0.0001$

An example of the impact of the choice of shape parameter $\beta$ on the reconstruction is shown in Fig. 5. Here, we used simulated MEG data generated by a patch of activity located in the occipital region of the left hemisphere. The same anatomical information and MEG sensors used for real dataset (cf. “MEG Real data” section) were used. A biological noise, simulated as in Calvetti et al. (2019), was added to the signal, yielding $SNR = 15$. It is clear that when $\eta =0.0001$ (see Fig. 5, bottom) the IAS algorithm favors sparse current density estimates respect to a choice of $\eta =0.1$ (see Fig. 5, middle) where the estimated density has a more spread distribution.

### MEG Real Data

**Fig. 6:**
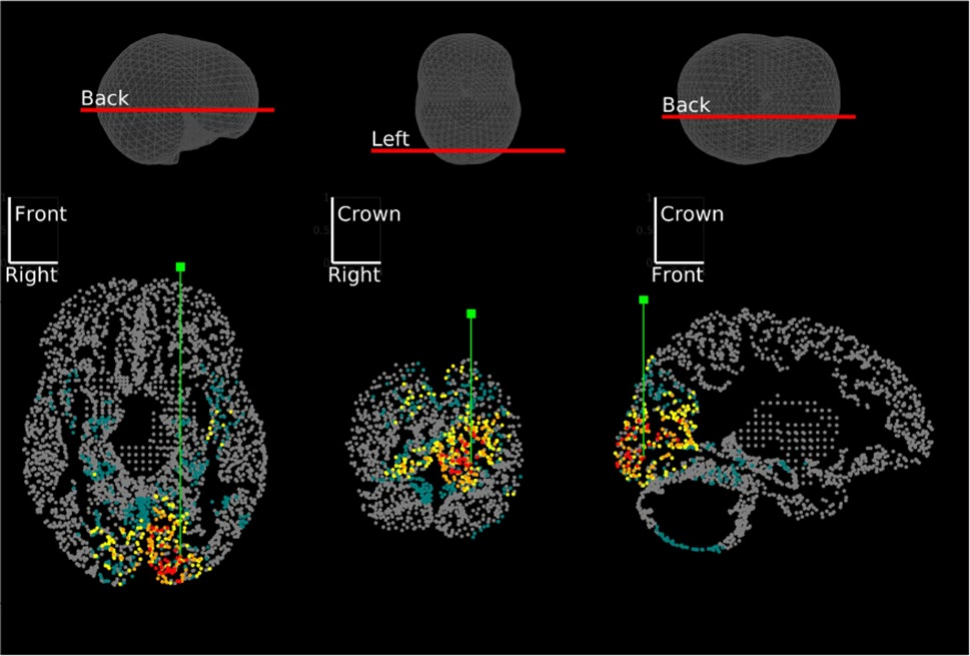
Visualization of the activity along slices passing through the maximal reconstructed activity

**Fig. 7:**
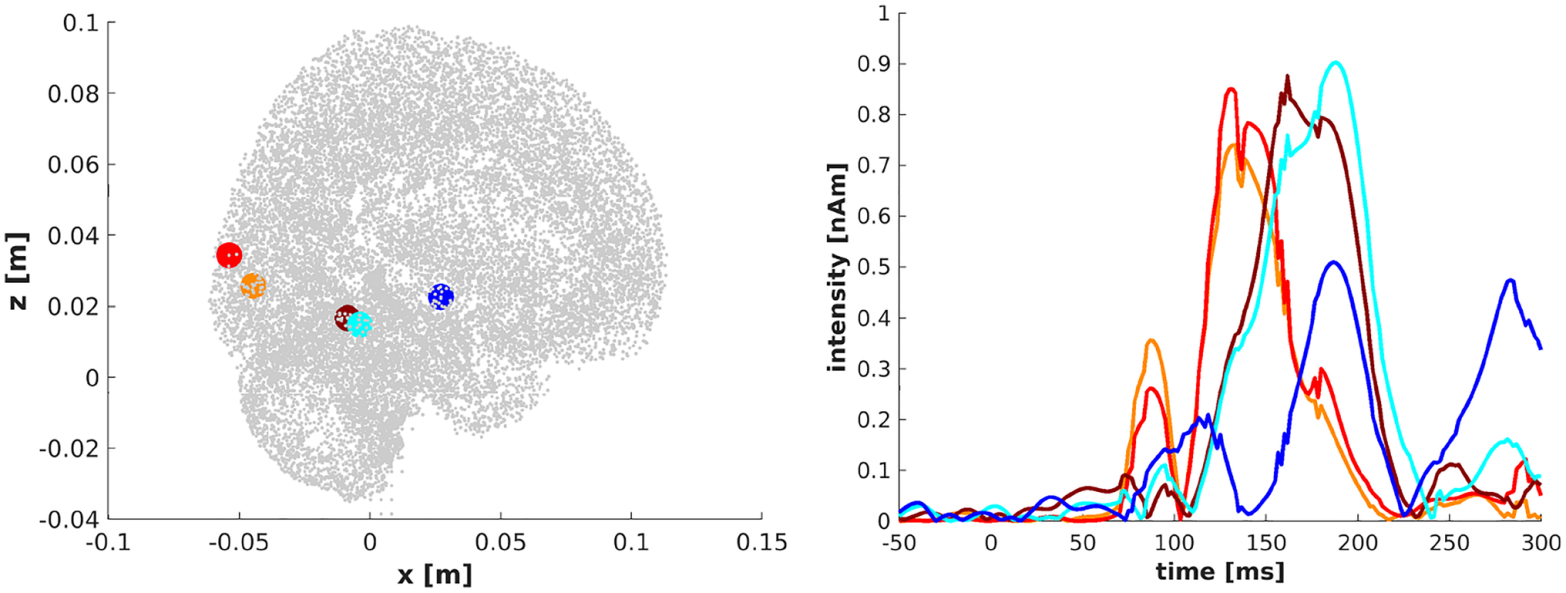
Time series of the intensity of the active dipoles

In the documentation (https://github.com/IAS-code/IAS-MEEG/blob/master/IAS_Demo.m) we provide an example of reconstruction obtained by applying IAS-MEEG algorithm to a real dataset. We used the MEG sample data provided by MNE software package (Gramfort et al. 2014) which includes also the MRI reconstructions created with FreeSurfer. These MEG data were acquired with the Neuromag Vectorview system at MGH/HMS/MIT Athinoula A. Martinos Center Biomedical Imaging. In the protocol experiment, checkerboard patterns were presented into the left and right visual field, interspersed by tones to the left or right ear. The interval between the stimuli was 750ms. Occasionally, a smiley face was presented at the center of the visual field. The subject was asked to press a key with the right index finger as soon as possible after the appearance of the face. In our example we only consider the trials corresponding to the left visual stimulus and perform the averaging on these trials. The MRI data were imported in Brainstorm (Tadel et al. 2011) to generate a source space including both the cortical surface and substructure regions. Following (Calvetti et al. 2019), we adopt for the source space the DBA (Deep Brain Activity) model proposed by Attal et al. (2012), Attal and Schwartz (2013) where the deep regions are modeled either with surfaces or volumes depending on anatomical information. The source space obtained in this manner consists of around 19000 nodes and the lead field matrix was computed using the single layer model implemented in OpenMEEG (Gramfort et al. 2010) software provided by Brainstorm. We reconstructed the neural activity by IAS-MEEG using the following input parameters: $SNR=9$, $\eta =0.01$ and $cut\_off=0.9$. Figure 6 shows the reconstructed activity for the left visual stimulus at 92ms where evidently an activation in the right occipital region appears. The visual stimulus elicits activity in different regions of the visual cortex, thus to get an overview in time of the different activated regions at different time points, the dipoles with maximum activity were selected and visualized. Figure 7 shows these reconstructed dipoles with the corresponding time trace.

### EEG Real Data

**Fig. 8:**
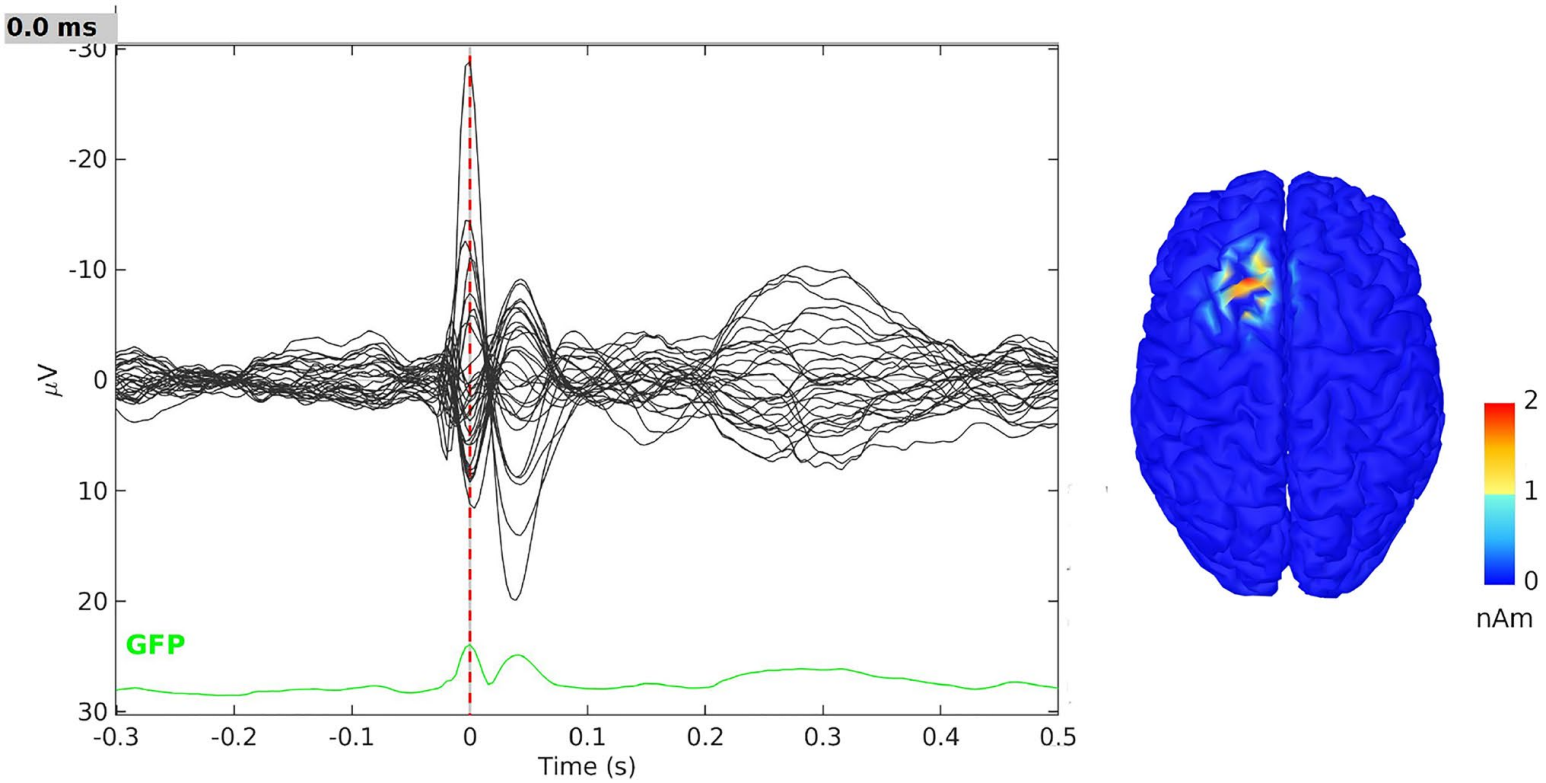
IAS localization on epileptic spikes from EEG real data

To show an example on how to use the IAS-MEEG algorithm within Brainstorm we run the plugin on the EEG sample dataset provided in the Tutorial EEG and Epilepsy https://neuroimage.usc.edu/brainstorm/Tutorials/Epilepsy. The tutorial dataset was acquired in a patient who suffered from focal epilepsy at the Epilepsy Center Freiburg, Germany. The EEG data was recorded at 256Hz, using a Neurofile NT digital video-EEG system with 128 channels and a 16-bit A/D converter. The spikes were marked with Brainstorm by the epileptologists at the Epilepsy Center in Freiburg. The individual MRI data were imported in Brainstorm and a cortical source space with around 15000 nodes was created. The lead field matrix was computed using a three layer model (scalp, skull and bran) by OpenMEEG software provided in Brainstorm. The IAS-MEEG algorithm was applied to the EEG data averaged on the marked spikes. Figure 8 shows the IAS localization on epileptic spikes when the following input parameters were used: $SNR=9$, $\eta =0.001$ and $cut\_off=0.9$. In particular, we used a value of $\eta$ that promotes focality.

## Conclusions

In this article we presented the IAS-MEEG package, a standalone, Matlab-based, freely downloadable software for the reconstruction of the neural activity from M/EEG data, with a plugin to integrate it in Brainstorm. The IAS-MEEG package is based on an Iterative Alternating Sequential (IAS) inversion algorithm that combines hierarchical Bayesian models and Krylov subspace iterative least squares solvers. The package is available via GitHub at https://github.com/IAS-code/IAS-MEEG and distributed under a Berkeley Software Distribution (BSD) license. An online documentation is also provided at https://ias-code.github.io/IAS-MEEG/index.html. All core routines are written in standard Matlab language and do not rely on any special packages. Furthermore, the inverse solver and visualization modules can be used either individually or in combination, allowing an easy integration in Brainstorm (Tadel et al. 2011).

## Data Availability

The MEG real data are available within the MNE software package. The EEG real data are available within Brainstorm. The authors thank the Epilepsy Centre in Freiburg for allowing to use the EEG dataset.

## Appendix

In this appendix, we review the scaling of the fields and matrices as implemented in the program *SetParameters*. To scale the lead field matrix, we write the action of the lead field matrix ${{\textsf{M}}}$ in terms of the single dipoles in the source space,

$$\begin{aligned} \big ({{\textsf{M}}}Q\big )_j = \sum _{k=1}^n (\textbf{p}_k^{(j)})^{{\textsf{T}}}\textbf{q}_k. \end{aligned}$$

Hence, the lead field vector $\textbf{p}^{(j)}_k$ indicates how much a unit dipole at position $\textbf{r}_k$ contributes to the *j*th channel. We define a scaling factor

$$\begin{aligned} {\overline{s}}^2 = \frac{1}{n} \sum _{k=1}^n \max _{1\le j\le m}\big \{\Vert \textbf{p}_k^{(j)}\Vert ^2\big \}, \end{aligned}$$

indicating the average effect over all dipoles on the channel that is most sensitive to a given dipole. To equilibrate numerically the lead field matrix, we write the forward model (1), indicating the time dependency with a subscript *t*, $1\le t\le T$, as

5 $$\begin{aligned} b_t= & {} {{\textsf{M}}}Q_t + \varepsilon = \left( \frac{1}{{\overline{s}}}{{\textsf{M}}}\right) ({\overline{s}} Q_t) + \varepsilon _t \nonumber \\= & {} {{\textsf{M}}}_{\textrm{sc}}({\overline{s}} Q_t) + \varepsilon _t, \end{aligned}$$

thus defining the scaled dimensionless lead field matrix ${{\textsf{M}}}_{\textrm{sc}}$. To scale the data, we define the mean amplitude over the time series,

$$\begin{aligned} {\overline{b}} = \frac{1}{T}\sum _{t=1}^T \Vert b_t\Vert , \end{aligned}$$

and scale the Eq. (5) to read

$$\begin{aligned} \frac{1}{{\overline{b}}} b_t = {{\textsf{M}}}_{sc}\left( \frac{{\overline{s}}}{{\overline{b}}}Q_t\right) + \frac{1}{{\overline{b}}}\varepsilon _t, \end{aligned}$$

or, using the evident notation,

6 $$\begin{aligned} b_{\textrm{sc},t} = {{\textsf{M}}}_{\textrm{sc}} Q_{\textrm{sc},t} + \varepsilon _{\textrm{sc},t}. \end{aligned}$$

The standard deviation $\sigma _{\textrm{sc}}$ of the scaled noise is estimated based on the signal-to-noise ratio, and the Eq. (6) is whitened by dividing it by $\sigma _{\textrm{sc}}$.

## References

[CR1] Aine CJ, Sanfratello L, Ranken D, Best E, MacArthur JA, Wallace T, Gilliam K, Donahue CH, Montano R, Bryant JE (2012). MEG-SIM: a web portal for testing MEG analysis methods using realistic simulated and empirical data. Neuroinformatics.

[CR2] Algorri ME, Flores-Mangas F (2004). Classification of anatomical structures in MR brain images using fuzzy parameters. IEEE Trans Biomed Eng.

[CR3] Auranen T, Nummenmaa A, Hämäläinen MS, Jääskeläinen IP, Lampinen J, Vehtari A, Sams M (2005). Bayesian analysis of the neuromagnetic inverse problem with $$\ell _p$$-norm priors. NeuroImage.

[CR4] Attal Y, Maess B, Friederici A, David O (2012). Head models and dynamic causal modeling of subcortical activity using magnetoencephalographic/electroencephalographic data. Rev Neurosci.

[CR5] Attal Y, Schwartz D (2013). Assessment of subcortical source localization using deep brain activity imaging model with minimum norm operators: a MEG study. PLoS ONE.

[CR6] Baillet S, Garnero L (1997). A Bayesian approach to introducing anatomo-functional priors in the EEG/MEG inverse problem. IEEE Trans BME.

[CR7] Baillet S, Mosher JC, Leahy RM (2001). Electromagnetic brain mapping. IEEE Signal Proc Mag.

[CR8] Bernardo JM, Smith AFM (2004). Bayesian theory.

[CR9] Brette R, Destexhe A (2012). Handbook of neural activity measurement.

[CR10] Calvetti D, Hakula H, Pursiainen S, Somersalo E (2009). Conditionally Gaussian hypermodels for cerebral source localization. SIAM J Imag Sci.

[CR11] Calvetti D, Pascarella A, Pitolli F, Somersalo E, Vantaggi B (2015). A hierarchical Krylov–Bayes iterative inverse solver for MEG with physiological preconditioning. Inverse Probl.

[CR12] Calvetti D, Pascarella A, Pitolli F, Somersalo E, Vantaggi B (2019). Brain activity mapping from meg data via a hierarchical Bayesian algorithm with automatic depth weighting. Brain Topogr.

[CR13] Calvetti D, Pitolli F, Somersalo E, Vantaggi B (2018) Bayes meets Krylov: preconditioning CGLS for underdetermined systems. SIAM Rev 60. 10.1137/15M1055061

[CR14] Calvetti D, Somersalo E, Strang A (2019) Hierachical Bayesian models and sparsity: $$\ell _2$$-magic Inverse Problems 35:035003

[CR15] Ciofolo C, Barillot C (2009). Atlas-based segmentation of 3D cerebral structures with competitive level sets and fuzzy control. Med Image Anal.

[CR16] Collins D Louis, Zijdenbos Alex P, Kollokian Vasken, Sled John G, Kabani Noor J, Holmes Colin J, Evans Alan C (1998). Design and construction of a realistic digital brain phantom. IEEE Trans BME.

[CR17] Dale AM, Fischl B, Sereno MI (1999). Cortical surface-based analysis: I. Segmentation and surface reconstruction. NeuroImage.

[CR18] Dale MA, Liu AK, Fischl BR, Buckner RL, Belliveau JW, Lewine JD, Halgren E (2000). Dynamic statistical parametric mapping: combining fMRI and MEG for high-resolution imaging of cortical activity. Neuron.

[CR19] de Munck JC, Vijn PCM, da Lopes SFH (1992). A random dipole model for spontaneous brain activity. IEEE Trans BME.

[CR20] Desikan RS, Ségonne F, Fischl B, Quinn BT, Dickerson BC, Blacker D, Buckner RL, Dale AM, Maguire RP, Hyman BT (2006). An automated labeling system for subdividing the human cerebral cortex on MRI scans into gyral based regions of interest. NeuroImage.

[CR21] Gorodnitsky IF, Rao BD (1997). Sparse signal reconstruction from limited data using FOCUSS: a re-weighted minimum norm algorithm. IEEE Trans. Signal Process.

[CR22] Gramfort A, Papadopoulos T, Olivi E, Clerc M (2010) Open MEEG: open source software for quasistatic bioelectromagnetics. Biomed Eng 10.1186/1475-925X-9-45 PMC2949879 20819204

[CR23] Gramfort A, Luessi M, Larson E, Engemann DA, Strohmeier D, Brodbeck C, Parkkonen L, Hämäläinen MS (2014). MNE software for processing MEG and EEG data. NeuroImage.

[CR24] Hedrich T, Pellegrino G, Kobayashi E, Lina JM, Grova G (2017). Comparison of the spatial resolution of source imaging techniques in high-density EEG and MEG. NeuroImage.

[CR25] Henson RN, Mattout J, Phillips C, Friston KJ (2009). Selecting forward models for MEG source-reconstruction using model-evidence. NeuroImage.

[CR26] Henson RN, Flandin G, Friston KJ, Mattout J (2010). A parametric empirical Bayesian framework for fMRI-constrained MEG/EEG source reconstruction. Hum Brain Mapp.

[CR27] Hämäläinen MS, Hari R, Ilmoniemi RJ, Knuutila J, Lounasmaa OV (1993). Magnetoencephalography-theory, instrumentation, and applications to noninvasive studies of the working human brain. Rev Mod Phys.

[CR28] Hämäläinen MS and Ilmoniemi RJ (1984) Interpreting measured magnetic fields of the brain: estimates of current distributions. Report TKK-F-A559

[CR29] Huizenga HM, JaC De Munck, Waldorp LJ, Grasman RPPP (2002). Spatiotemporal EEG/MEG source analysis based on a parametric noise covariance model. IEEE Trans BME.

[CR30] Kass RE, Raftery AE (1995). Bayes factors. JASA.

[CR31] Kiebel SJ, Daunizeau J, Phillips C, Friston KJ (2008). Variational Bayesian inversion of the equivalent current dipole model in EEG/MEG. NeuroImage.

[CR32] Kybic J, Clerc M, Abboud T, Faugeras O, Keriven R, Papadopoulos T (2005). A common formalism for the integral formulations of the forward EEG problem. IEEE Trans Med Imaging.

[CR33] Lin FH, Witzel T, Ahlfors SP, Stufflebeam SM, Belliveau JW, Hämäläinen MS (2006). Assessing and improving the spatial accuracy in MEG source localization by depth-weighted minimum-norm estimates. NeuroImage.

[CR34] Lin FH, Belliveau JW, Dale AM, Hämäläinen MS (2006). Distributed current estimates using cortical orientation constraints. Hum Brain Mapp.

[CR35] Lopez JD, Litvak V, Espinosa JJ, Friston K, Barnes GR (2014). Algorithmic procedures for Bayesian MEG/EEG source reconstruction in SPM. NeuroImage.

[CR36] Lucka F, Pursiainen S, Burger M, Wolters CH (2012). Hierarchical Bayesian inference for the EEG inverse problem using realistic FE head models: depth localization and source separation for focal primary currents. NeuroImage.

[CR37] Mattout J, Phillips C, Penny WD, Rugg MD, Friston KJ (2006). MEG source localization under multiple constraints: an extended Bayesian framework. NeuroImage.

[CR38] Molins A, Stufflebeam SM, Brown EN, Hämáläinen MS (2008). Quantification of the benefit from integrating MEG and EEG data in minimum? 2-norm estimation. Neuroimage.

[CR39] Mosher JC, Spencer ME, Leahy RM, Lewis PS (1993). Error bounds for EEG and MEG dipole source localization. Electroenceph Clin Neurophysiol.

[CR40] Nagarajan SS, Portniaguine O, Hwang D, Johnson C, Sekihara K (2006). Controlled support MEG imaging. NeuroImage.

[CR41] Nummenmaa A, Auranen T, Hämäläinen MS, Jääskeläinen IP, Lampinen J, Sams M, Vehtari A (2007). Hierarchical Bayesian estimates of distributed MEG sources: theoretical aspects and comparison of variational and MCMC methods. NeuroImage.

[CR42] Nummenmaa A, Auranen T, Hämäläinen MS, Jääskeläinen IP, Lampinen J, Sams M, Vehtari A (2007). Automatic relevance determination based hierarchical Bayesian MEG inversion in practice. NeuroImage.

[CR43] Oostenveld R, Fries P, Maris E, Schoffelen JM (2011) FieldTrip: open source software for advanced analysis of MEG, EEG, and invasive electrophysiological data. Comput Intell Neurosci 10.1155/2011/156869 PMC3021840 21253357

[CR44] Owen JP, Wipf DP, Attias HT, Sekihara K, Nagarajan SS (2012). Performance evaluation of the Champagne source reconstruction algorithm on simulated and real M/EEG data. NeuroImage.

[CR45] Parkkonen L, Fujiki N, Mäkelä JP (2009). Sources of auditory brainstem responses revisited: contribution by magnetoencephalography. Hum Brain Mapp.

[CR46] Pascual-Marqui RD (1999). Review of methods for solving the EEG inverse problem. Int J Bioelectromagn.

[CR47] Sato M, Yoshioka T, Kajihara S, Toyama K, Goda N, Doya K, Kawato M (2004). Hierarchical Bayesian estimation for MEG inverse problem. NeuroImage.

[CR48] Stephan KE, Penny WD, Daunizeau J, Moran RJ, Friston KJ (2009). Bayesian model selection for group studies. NeuroImage.

[CR49] Tadel F, Baillet S, Mosher JC, Pantazis D, Leahy RM (2011). Brainstorm: a user-friendly application for MEG/EEG analysis. Comp Intell Neurosci.

[CR50] Trujillo-Barreto NJ, Aubert-Vázquez E, Valdés-Sosa PA (2004). Bayesian model averaging in EEG/MEG imaging. NeuroImage.

[CR51] Uutela K, Hämäläinen MS, Somersalo E (1999). Visualization of magnetoencephalographic data using minimum current estimates. NeuroImage.

[CR52] Wagner M, Fuchs M, Kastner J (2004). Evaluation of sLORETA in the presence of noise and multiple sources. Brain Topogr.

[CR53] Wipf D, Nagarajan S (2009). A unified Bayesian framework for MEG/EEG source imaging. NeuroImage.

[CR54] Wipf DP, Owen JP, Attias HT, Sekihara K, Nagarajan SS (2010). Robust Bayesian estimation of the location, orientation, and time course of multiple correlated neural sources using MEG. NeuroImage.

[CR55] Vorwerk J, Cho JH, Rampp S, Hamer H, Knösche TR, Wolters CH (2014). A guideline for head volume conductor modeling in EEG and MEG. NeuroImage.

